# Physical Deterioration

**Published:** 1904-08-06

**Authors:** 


### The Hospital.

A Journal of

The Medical Sciences and Hospital Administration.

Vol. XXXVI.—No. 932. AUGUST 6, 1904.

### Physical Deterioration.

The voluminous report issued by the committee appointed to inquire into the question of physical deterioration is eminently creditable to the good sense of its authors, and practically embodies the conclusions at which persons fairly conversant with the general conditions of our population had in most cases arrived. The committee are to a great extent debarred from forming positive conclusions by the absence of any trustworthy materials for comparison between the present and the past; but they are unable to find any convincing evidence of physical deterioration as a general national condition. They admit that large strata of the people are in a state that leaves much to be desired, and they enter at great length into the probable causes of the amount of depressed vitality which they cannot refuse to recognise, and into the remedies by which it is possible that a better state of things might be brought about. They give the first place to measures for the removal of the uncertainty concerning facts which now exists; and under this head they propose that all children attending rate-supported schools, and all children engaged in work which brings them under the cognisance of certifying surgeons under the Factory Acts, should be periodically weighed and measured, and their weights and measurements recorded for future reference. They suggest that these observations, and the manner of taking them, should be under the general control and supervision of a Central Board of skilled persons, but that the actual work should be performed, in every locality, by the teachers and medical practitioners available in the district, and that districts should be so framed, as far as possible, as to include only fairly homogeneous populations. The necessary weighings and measurings, if conducted annually, would at the end of ten years afford convincing evidence of any change, either for the better or for the worse, which had occurred during the interval in the juvenile local population generally; and would also afford a continuous means of watching and testing the progress of individual children and families. A procedure which was enforced by law upon the children over whom the law would have control, and which was manifestly calculated to be of great public advantage, would in all probability be voluntarily adopted in the great majority of private schools; and, if this expectation were fulfilled, the facts about the condition of the people, in all classes of life, would speedily be rendered accessible to scientific analysis. We should no longer be left in doubt with regard either to the facts themselves, or to the remedies which were indicated for any imperfections or shortcomings which might be disclosed.

There can, we think, be very little doubt that the systematic inquiry recommended by the Committee would soon attach physical deficiency to two sets of easily recognisable circumstances. It would be found in *families* in which the parents, either from poverty, from ignorance, or from neglect, exposed their children to the continued operation of unfavourable conditions; and it would be found in *districts* in which the local authorities were remiss in the discharge of their duties concerning local sanitation. The neglected or poverty-stricken families might be dealt with either by the law or by charity, as the facts and the local circumstances might dictate; but it would probably be desirable, at least in extreme cases, to provide some means by which criminally-neglected children could be removed entirely from the parental control, although the parents should by no means be relieved from responsibility for the cost of their proper maintenance. The neglected districts would call for the supersession of the guilty authorities by the medical officer of health for the county, or by the Local Government Board; and this supersession should be accomplished in such a way as to attach public and manifest disgrace to the delinquents, and, if possible, to inflict some kind of direct pecuniary penalty upon the ratepayers or others by whom they had been elected. In this way, and probably in this way only, it would be possible to check the present practice of placing the proprietors of nuisances upon public boards, where their sole object is to prevent these nuisances from being abated.

The committee, as was to be expected, lay great stress upon alcohol and tobacco as causes of physical deterioration; and they say, among other things, that the effect upon offspring of maternal drunkenness, or, at least, of the drunkenness of both parents, is very much greater and more serious than that of the drunkenness of the father only, and also that drunkenness among married women is greatly upon the increase. Their indictment of tobacco is mainly based upon its assumed directly deleterious influence upon young smokers; and no ascertained or probable effect upon offspring is even referred to. However, nothing is more certain than the frequent transmission of an unstable nervous system to offspring; and few things seem more calculated to produce such instability than the daily saturation with a narcotic of the body of an undernourished and intemperate man. We are disposed to think that a complete examination into the effects of tobacco on the population would justify a much more serious indictment than that for which the committee has become responsible.

